# Nanotechnology for the management of COVID-19 during the pandemic and in the post-pandemic era

**DOI:** 10.1093/nsr/nwac124

**Published:** 2022-06-27

**Authors:** Chun Xu, Chang Lei, Sepanta Hosseinpour, Saso Ivanovski, Laurence J Walsh, Ali Khademhosseini

**Affiliations:** School of Dentistry, The University of Queensland, Brisbane 4006, Australia; Centre for Orofacial Regeneration, Reconstruction and Rehabilitation (COR3), School of Dentistry, The University of Queensland, Brisbane 4006, Australia; Australian Institute for Bioengineering and Nanotechnology, The University of Queensland, St Lucia 4072, Australia; School of Dentistry, The University of Queensland, Brisbane 4006, Australia; Centre for Orofacial Regeneration, Reconstruction and Rehabilitation (COR3), School of Dentistry, The University of Queensland, Brisbane 4006, Australia; School of Dentistry, The University of Queensland, Brisbane 4006, Australia; Centre for Orofacial Regeneration, Reconstruction and Rehabilitation (COR3), School of Dentistry, The University of Queensland, Brisbane 4006, Australia; School of Dentistry, The University of Queensland, Brisbane 4006, Australia; Terasaki Institute for Biomedical Innovation, Los Angeles, CA 90064, USA

**Keywords:** COVID-19, nanotechnology, SARS-CoV-2, diagnosis, vaccines

## Abstract

Following the global COVID-19 pandemic, nanotechnology has been at the forefront of research efforts and enables the fast development of diagnostic tools, vaccines and antiviral treatment for this novel virus (SARS-CoV-2). In this review, we first summarize nanotechnology with regard to the detection of SARS-CoV-2, including nanoparticle-based techniques such as rapid antigen testing, and nanopore-based sequencing and sensing techniques. Then we investigate nanotechnology as it applies to the development of COVID-19 vaccines and anti-SARS-CoV-2 nanomaterials. We also highlight nanotechnology for the post-pandemic era, by providing tools for the battle with SARS-CoV-2 variants and for enhancing the global distribution of vaccines. Nanotechnology not only contributes to the management of the ongoing COVID-19 pandemic but also provides platforms for the prevention, rapid diagnosis, vaccines and antiviral drugs of possible future virus outbreaks.

## Teaser

This review highlights how nanotechnology helps us in the management of COVID-19 by providing detection methods, vaccines and antiviral treatments during the pandemic and in the post-pandemic era.

## INTRODUCTION

The current global coronavirus disease 2019 (COVID-19) pandemic caused by the novel coronavirus (severe acute respiratory syndrome coronavirus 2, SARS-CoV-2) that began in 2019 has greatly changed the world. According to the World Health Organization (WHO), the number of confirmed cases has exceeded 418 million, with over 5.8 million deaths across over 200 countries and territories (as of February 2022) [1].

In the battle against surges in infection with SARS-CoV-2, nanotechnology has been at the forefront, enabling the rapid development of diagnostic methods, vaccines and antiviral medicines [2,3]. Nanoparticle-based detection methods such as lateral flow assays (LFAs) for SARS-CoV-2 antigens have been used for rapid antigen testing. Nanopore-based sequencing platforms have provided alternatives for molecular genetic analyses using the real-time reverse transcription-polymerase chain reaction (RT-PCR) [4]. Antiviral nanomaterials are also emerging as pandemic countermeasures [5]. Nanoparticles have been prominent in mRNA vaccine designs, where lipid nanoparticles have been used to deliver mRNA in the vaccines from Pfizer and Moderna, with over 10 billion doses having been administered within the first two years of the pandemic.

At the global level, the struggle with containing the virus continues, as more contagious variants have emerged, such as the Delta (B.1.617.2) and Omicron strains (B.1.1.529). The Omicron variant spread at unprecedented speed, with over 125 million new infections each day during January 2022, which was 10 times faster than Delta. With rising levels of COVID-19 vaccination, including the use of booster third vaccine doses, combined with high levels of infection-acquired immunity, some observers have predicted the end of the pandemic in 2022, but with SARS-CoV-2 continuing as a fifth endemic human coronavirus in global circulation for the foreseeable future [6]. Thus, the world's population will be living with the virus in the post-pandemic era.

Nanotechnology will likely continue to contribute to the management of COVID-19-related issues, including the multiple health problems of ‘long haul COVID’ that develop more than three months after the acute infection. In the post-pandemic era, the application of nanotechnology is important in terms of global preparation for future viral pandemics. In this review, we discuss the role of nanotechnology in the diagnosis and treatment of COVID-19 and its role in the post-pandemic era. We discuss nanotechnology as applied to the detection of SARS-CoV-2, vaccine development and the development of antiviral medicines. From there, we discuss the role of nanotechnology in the post-pandemic era and highlight nanoscale information in the battle with SARS-CoV-2 variants and the global distribution of vaccines. Finally, we present our perspectives on nanotechnology for the management of viral diseases, considering the lessons learnt that will inform the management of future pandemics, in terms of nanotechnology for prevention, diagnosis and treatment.

## NANOTECHNOLOGY FOR DETECTION OF SARS-COV-2

Rapid identification of infected patients is essential for effective isolation and quarantine protocols, to help limit the spread of infection. Within weeks after the first published report of COVID-19, the full sequence of the SARS-CoV-2 genome had been determined, and it was shared globally, informing the development of vaccines and medicines. Nanoscale features of the SARS-CoV-2 virus were characterized using cryo-electron microscopy [7,8]. These analyses revealed that for viral entry into human cells, the viral spike (S) protein binds to angiotensin-converting enzyme 2 (ACE2), and this is followed by membrane fusion of the viral envelope and the host cell membrane.

**Figure 1.**
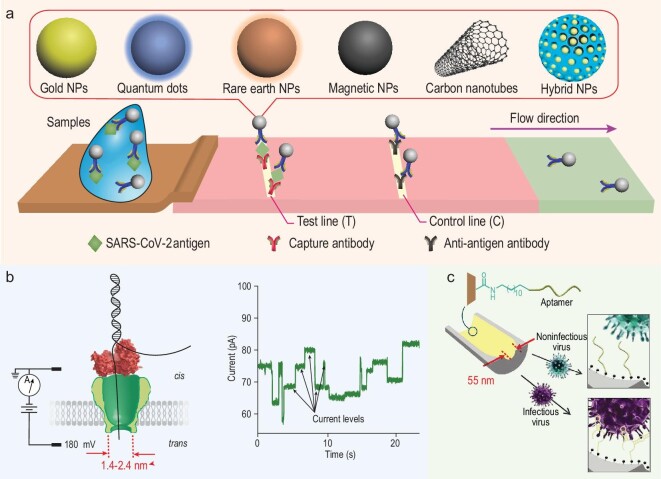
Nanotechnologies used for detection of SARS-CoV-2. (a) Nanoparticle-based rapid antigen tests use various nanoengineered approaches, including colloidal gold nanoparticles, quantum dots, rare earth nanoparticles (upconversion and downconversion nanoparticles), magnetic nanoparticles, carbon nanotubes and hybrid nanoparticles with sizes ranging from several to several hundred nanometers. (b) The nanopore-based sequencing technique for virus detection. (c) A porous nanomaterial-based sensor for virus detection. Panel b reproduced with permission from ref. [16]. Panel c reproduced with permission from ref. [9]. NPs = nanoparticles.

Traditional immunoassays and nucleic-acid-amplification tests using RT-PCR have been deployed to measure antibody levels and viral load, to assess past infection and current infection, respectively. Due to high levels of demand for these laboratory-based tests, greater emphasis has been placed on point-of-care and self-administered rapid antigen testing (RAT). Such testing has allowed timely identification of asymptomatic cases, in workplaces, in the general community and in critical services such as health care. RAT uses nanoparticles, particularly colloidal gold (Fig. 1a), for generating a visual change. For nucleic acid detection, in addition to traditional RT-PCR, nanopore-based sequencing techniques for virus detection can also be used (Fig. 1b). Nanomaterial-based sensors for virus detection have also been developed (Fig. 1c) [9].

### Nanoparticle-based techniques for detection of SARS-CoV-2

A range of nanoparticles, including colloidal gold nanoparticles, quantum dots (QDs), rare earth nanoparticles, magnetic nanoparticles, carbon nanotubes and hybrid nanoparticles such as QD-doped mesoporous silica nanoparticles, can be used in immunoassays to detect various targets (Fig. 1a) [10,11]. The tests can be used with nasal swabs, throat swabs, sputum samples, saliva samples or serum. There are 49 US FDA-approved antigen diagnostic devices for COVID-19, as of 28 May 2022, under emergency-use authorizations. Most of these diagnostic devices are LFA that use colloidal gold nanoparticles or QDs. In immunological assays, various nanoparticles are conjugated with antibodies, to either reveal a visible colour change or to allow fluorescent/electrochemical/magnetic signal detection when the conjugated antibody has bound to the antigen. Colloidal-gold-nanoparticle-based LFA is the most common one, with the advantages of low cost and a simple result-reading method (just by sight; no instrument is needed). The fluorescence-based detection method uses fluorophores such as QDs and rare earth nanoparticles (upconversion and downconversion nanoparticles) and offers higher sensitivity and a lower detection limit compared to colourimetric detection. QD-based fluorescence assays can achieve a sensitivity at least 10 times higher than gold-based ones due to the lower background and higher brightness. However, special fluoresce reading instruments are needed for the results reading. The strategy of using a smart phone’s camera with a simple light source may provide a cheap and simple method of fluorescence-based detection. Magnetic LFA provides another strategy for antigens tests, by measuring the stray field changes from the magnetic nanoparticles. These methods offer very high sensitivity and low detection limits due to no or negligible background noise. However, special instruments such as giant magnetoresistance sensors are needed for the detection and are thus not widely used yet. Recently, hybrid nanoparticles such as QD-loaded mesoporoussilica-based LFA could improve detection sensitivity by 10^4^ times compared to commercial colloidal-gold-based LFA, and thus may be used for early detection of SARS-CoV-2 infection [11].

To detect the SARS-CoV-2 virus in an LFA, the primary targets are the spike (S) protein, the nucleocapsid (N) protein of the virus, the antibodies or the viral nucleic acid [12]. N protein antigen-detecting LFAs have superior sensitivity over traditional serological assays, with a limit of detection (LOD) of 3.03 ng/mL [13]. To exploit the widespread availability of smartphones and the high quality of cameras in smartphones, a novel assay has been developed that uses a nano-enzyme-based chemiluminescence process to detect the S protein. This assay has an LOD of 0.1 ng/mL [14].

RAT has been deployed on a massive scale in many countries, and the cost per test is low. The trade-off for RAT is that it is less sensitive than expensive laboratory assays, by a factor of 10^5^ times compared to RT-PCR, and by 10^3^ times for cell culture assays [15]. One way of reducing this performance gap in terms of sensitivity is to use multiplex approaches and fluorescence detection for immunoassays, rather than simple visual readouts.

### Nanopore-based techniques for detection of SARS-CoV-2

#### Nanopore sequencing

Nanopore sequencing allows real-time analysis of long DNA or RNA fragments by using voltage-biased nanoscale pores in a membrane [16,17]. By engineering protein nanopores with a 5 nm long stem and an inside channel diameter that varies from ∼1.4 to ∼2.4 nm [18,19], the passage of a linear, single-stranded (ss) DNA or RNA molecule through that pore can cause changes in current flow (Fig. 1b). The measurement of these changes can be decoded using an algorithm to generate a nucleic acid sequence [16]. This nanopore sequencing technique was used to sequence the transcriptome of SARS-CoV-2 [20], and then the full-length genomic RNA of the virus [21].

Bull *et al*. evaluated the analytical performance of nanopore sequencing for COVID-19 using the sequencing devices from Oxford Nanopore Technologies, and specimens from SARS-CoV-2-positive patients (total number: 157) and synthetic RNA controls. The results showed highly accurate consensus-level sequence determination, with >99% sensitivity and >99% precision with regard to single nucleotide variants detected above a minimum ∼60-fold coverage depth. This study demonstrates the value of nanopore sequencing for SARS-CoV-2 genome analysis. However, it was also reported that this technique failed to detect variants at low read-count frequencies accurately [4]. Likewise, Wang *et al*. combined real-time nanopore sequencing with targeted amplification methods for the detection of SARS-CoV-2. Within 6–10 hours, this approach can detect and categorize SARS-CoV-2 variants and other respiratory viruses. Clinical diagnostic tests have validated this technique [22].

#### Nanomaterial-based sensing

Sensors based on nanomaterials, especially porous nanomaterials, have been developed for the direct detection of infectious virions of SARS-CoV-2. DNA aptamers, which bind intact infectious virions (but not non-infectious virions), have been incorporated into solid-state nano-sized pores. This allows the nanomaterial to strongly bind and confine the virus, which increases the sensitivity and lowers the detection limit down to 10 000 copies/mL for SARS-CoV-2, and 1 pfu/mL for human adenovirus (Fig. 1c) [9]. These combined aptamer-nanopore sensors have been used with different sample types, including water, saliva and serum, for the detection of both enveloped and non-enveloped viruses. Aptamer-nanopore sensors have broad application when it comes to detecting viruses of public health concern. More recently, metal-organic framework (MOF)-based sensors have also been developed for SARS-CoV-2 detection; these utilize the porous structure and high surface area of MOFs. Rabiee and co-authors synthesized MOF-5 with CoNi_2_S_4_ (for enhancing the selectivity of sensors) and decorated it with the porphyrin, H2TMP (as a sensitizer for sensors) and achieved a detection limit of 5 nM for the SARS-CoV-2 spike antigen [23]. This proof-of-concept study demonstrates the potential of using MOFs as low-cost and efficient sensors for COVID-19 detection.

In addition to single-plexed detection, multiplexed sensing of SARS-CoV-2 has also been recently developed based on nanomaterials. Gao and his group designed a multiplexed and wireless electrochemical platform based on graphene for ultra-rapid detection of COVID-19 [24,25]. This nanomaterial-based detection technique can test SARS-CoV-2 antigens, antibodies and C-reactive proteins to provide key information, including the viral infection, immune response and disease severity. They also validated this platform using COVID-19 patient blood and saliva samples.

## NANOTECHNOLOGY AND COVID-19 VACCINES

Vaccines remain the most efficient strategy for protection against viral infections. An appropriate vaccine will generate antibodies and memory T cells as well as sensitized cytotoxic cells. Vaccine design includes the identification of antigens and adjuvants, as well as an appropriate delivery method to deliver the antigens and trigger proper immune reactions. Nanotechnology provides powerful tools to deliver the antigens and present them to the immune system, and also acts like adjuvants to elicit stronger immune responses. During this pandemic, the Pfizer and Moderna mRNA vaccines are using lipid nanoparticles to deliver the mRNA that encodes for the spike protein of SARS-CoV-2, and these represent a milestone for both nanotechnology and the mRNA technique. The mRNA induces the expression of the spike protein, triggering the host immune response. Both the Pfizer and Moderna mRNA vaccines have a reported efficacy of around 95% for preventing hospitalizations caused by laboratory-confirmed COVID-19 in people aged 16 or older who have not been previously infected with SARS-CoV-2 [26–28].

**Figure 2.**
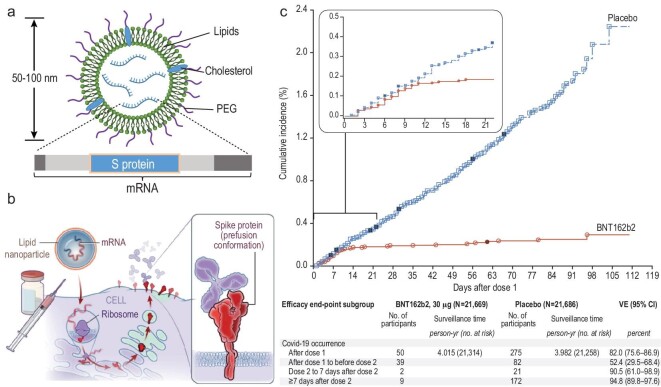
Lipid nanoparticle-based mRNA vaccines for COVID-19. (a) A schematic of the structure of an mRNA lipid nanoparticle vaccine. (b) The immune response to the mRNA vaccine. (c) The efficacy of the Pfizer mRNA vaccine for COVID-19 (red) versus a placebo (blue), with the cumulative incidence over four months after the first injection. Panel b and c reproduced with permission from ref. [26].

As shown in Fig. 2, the mRNA is encapsulated in lipid nanoparticles comprised of lipid, cholesterol and polyethylene glycol (PEG). Since naked mRNA is not stable and is degraded readily by extracellular RNase enzymes, the lipid nanoparticle protects the mRNA during storage and after administration, and is essential for vaccine efficacy [29,30]. Cells internalize the nanoparticles, and the mRNA uses the normal cellular machinery for translation into proteins [31]. These lipid nanoparticles range in size between 80 and 200 nm and are synthesized by the self-assembly of cationic lipids [32].

In addition to lipid nanoparticles, other types of nanoparticles can be used as nanocarriers to deliver nucleic acids for vaccines, including PEG-lipid functionalized dendrimer nanoparticles, cationic peptides, cationic polymer nanoparticles and polyethyleneimine nanoparticles [33,34].

Nanoparticles can possess their own immunogenic properties, which can stimulate protective immune responses against coronaviruses and other viruses [35]. Relevant examples include gold nanoparticles, polymeric nanoparticles and spike protein nanoparticles [36]. Such particles could be administered orally, intranasally, and by subcutaneous or intramuscular injection. These alternative administration methods overcome tissue barriers and result in greater uptake of the particles into regional lymph nodes [37,38].

In addition to serving as a delivery system, nanoparticles can also be used as adjuvants, to boost the overall efficiency of the immune response generated by vaccines. Adjuvants also reduce the required antigen dose [39]. Of the various COVID-19 vaccines in development, 10 candidate vaccines have used protein subunits in combination with a nanoparticle adjuvant (6 at preclinical and 4 at clinical testing stage).

Vaccination using protein subunits also involves nanotechnology. These protein subunits comprise various external structural components of the SARS-CoV-2 virus and are combined with adjuvants to increase the extent of stimulation of the immune response [40]. An example of novel design is the so-called ‘molecular clamp’ approach, where the relevant subunit of the virus is confined in a nano-sized ‘clamp’ to keep the protein or peptide in the correct configuration [41].

Nanoparticles can be potent adjuvants in vaccines because of their immune-stimulating activity. They also have modifiable surface chemistry [42,43]. As an example, alumina nanoparticles coated with antigen have been shown to enhance humoral and cellular immune responses, because they facilitate antigen cross-presentation to T cells, and induce autophagy in dendritic cells [44]. Likewise, gold nanoparticles can drive increased expression of inflammatory cytokines [45]. Table 1 provides an overview of the landscape of nanotechnology-based vaccines that are under preclinical/clinical investigations, based on the information compiled in the WHO COVID-19 vaccine tracker, as of 16 May 2022 [46–49]. In total, there are 198 vaccines in the preclinical stage and 156 vaccines under clinical investigation. Among these vaccines, at least 69 have been developed with nanotechnology (41 preclinical development and 28 clinical development). So far, 10 vaccines have been granted emergency use listing (EUL) by the WHO (16 May 2022).

**Table 1.** The landscape of nano-assisted coronavirus vaccine candidates in clinical and pre-clinical trials.

Pre-clinical development		
Vaccine platform	Type of vaccine	Developer
Protein subunit	Nanoparticle vaccine	LakePharma, Inc.
	Spike-based	Nanografi Nano Technology, Middle East Technical University, Ankara University
	RBD protein delivered in mannose-conjugated chitosan nanoparticle	Ohio State University/Kazakh National Agrarian University
	Recombinant protein, nanoparticles (based on S-protein and other epitopes)	Saint Petersburg Scientific Research Institute of Vaccines and Serums
	Protein subunit nano-formulated	Vaxinano, CEA, INRAE
	Peptide antigens formulated in lipid nanoparticles (LNPs)	IMV Inc.
	Nanoparticle vaccine	LakePharma, Inc.
	S subunit intranasal liposomal formulation with GLA/3M052 adjs.	University of Virginia
RNA-based vaccine	LNP-encapsulated mRNA encoding S	Max Planck Institute of Colloids and Interfaces
	LNP-mRNA	Translate Bio/Sanofi Pasteur
	LNP-mRNA	CanSino Biologics/Precision NanoSystems
	LNP-encapsulated mRNA cocktail encoding virus-like particle (VLP)	Fudan University/Shanghai JiaoTong University/RNACure Biopharma
	LNP-encapsulated mRNA encoding RBD	Fudan University/Shanghai JiaoTong University/RNACure Biopharma
	LNP-encapsulated mRNA	University of Tokyo/Daiichi-Sankyo
	D614G variant LNP-encapsulated mRNA	Globe Biotech Ltd
	ZIP1642–a self-amplifying RNA (saRNA) vaccine encapsulated in an LNP, which encodes for multiple antigens, including the Spike (S) protein.	Ziphius Vaccines and Ghent University
	LNP-mRNA	Certest Biotec
	Liposome-encapsulated mRNA	BIOCAD
	mRNA	Selcuk University
	Several mRNA candidates	RNAimmune, Inc.
	mRNA	FBRI SRC VB VECTOR, Rospotrebnadzor, Koltsovo
	mRNA	China CDC/Tongji University/Stermina
	mRNA in an intranasal delivery system	eTheRNA
	mRNA	Greenlight Biosciences
	mRNA	IDIBAPS Hospital Clinic, Spain
	mRNA	Providence Therapeutics
	mRNA	Cell Tech Pharmed
	mRNA	ReNAP Co.
DNA-based vaccine	Plasmid DNA, nanostructured RBD	National Institute of Chemistry, Slovenia
Virus-like particle	Enveloped virus-like particle (eVLP)	VBI Vaccines Inc.
	S protein integrated in HIV VLPs	IrsiCaixa AIDS Research/IRTA-CReSA/Barcelona Supercomputing Centre/Grifols
	VLP + Adjuvant	Mahidol University/The Government Pharmaceutical Organization (GPO)/Siriraj Hospital
	VLPs, lentivirus and baculovirus vehicles	Navarrabiomed, Oncoimmunology group
	VLP, based on RBD displayed on VLPs	Saiba GmbH
	ADDomerTM multiepitope display	Imophoron Ltd and Bristol University's Max Planck Centre
	VLP	OSIVAX
	eVLP	ARTES Biotechnology
	VLPs peptides/whole virus	University of Sao Paulo
	VLPs produced in BEVS	Tampere University
	Plant-derived VLP	Shiraz University
	Plasmid-driven production of VLPs containing S, M, N and E proteins of SARS-CoV-2	Arizona State University
Protein subunit	SARS-CoV-2 rS/Matrix M1-adjuvant (full-length recombinant SARS-CoV-2 glycoprotein nanoparticle vaccine adjuvanted with Matrix M) NVX-CoV2373	Novavax	Phase 3	90.4% (CI 82.9–94.6)— 1 dose	[46]
	Recombinant SARS-CoV-2 Spike protein, aluminium adjuvanted (NanoCovax)	Nanogen Pharmaceutical Biotechnology	Phase 3	No report yet	-
	SpFN (spike ferritin nanoparticle)—uses spike proteins with a liposomal formulation QS21 (ALFQ) adjuvant	Walter Reed Army Institute of Research (WRAIR)	Phase 1	No report yet	-
	T-cell-priming specific cocktail of coronavirus peptides mounted on a gold nanoparticle	Emergex Vaccines	Phase 1	No report yet	-
RNA-based vaccine	CoV2 SAM LNP vaccine. A self-amplifying mRNA LNP platform + Spike antigen	GlaxoSmithKline	Phase 1	No report yet	-
	mRNA-1273 Spikevax	Moderna and National Institute of Allergy and Infectious Diseases (NIAID)	Phase 4	93.2% (CI 91.0 to 94.8)—2 doses	[47]
	ChulaCov19 mRNA vaccine	Chulalongkorn University	Phase 1/2	No report yet	-
	PTX-COVID19-B, mRNA vaccine	Providence Therapeutics	Phase 2	No report yet	-
	Chimpanzee Adenovirus serotype 68 (ChAd) and self-amplifying mRNA vectors expressing spike alone, or spike plus additional SARS-CoV-2 T cell epitopes	Gritstone Oncology	Phase 1	No report yet	-
	MRT5500, an mRNA vaccine candidate	Sanofi Pasteur and Translate Bio	Phase 2	No report yet	-
	mRNA-1283.211	ModernaTX, Inc.	Phase 1	No report yet	-
	mRNA COVID-19 vaccine	Shanghai East Hospital and Stemirna Therapeutics	Phase 1	No report yet	-
	ARCT-154 mRNA vaccine	Arcturus Therapeutics, Inc.	Phase 3	No report yet	-
	ARCT-165 mRNA vaccine	Arcturus Therapeutics, Inc.	Phase 1/2	No report yet	-
	ARCT-021 mRNA vaccine	Arcturus Therapeutics, Inc.	Phase 1/2	No report yet	-
	Coronavirus mRNA vaccine (LVRNA009)	AIM Vaccine and Liverna Therapeutics	Phase 2	No report yet	-
	mRNA-1273.529–Booster	ModernaTX, Inc.	Phase 2/3	No report yet	-
	CV2CoV, mRNA vaccine	CureVac AG	Phase 1	No report yet	-
	mRNA vaccine (MIPSCo-mRNA-RBD-1)	University of Melbourne	Phase 1	No report yet	-
	A Lyophilized COVID-19 mRNA vaccine	Jiangsu Rec-Biotechnology Co., Ltd	Phase 1	No report yet	-
	COVID-19 mRNA vaccine (SYS6006)	CSPC ZhongQi Pharmaceutical Technology Co., Ltd	Phase 1	No report yet	-
	HDT-301: self-replicating mRNA vaccine formulated as an LNP	SENAI CIMATEC	Phase 1	No report yet	-
	mRNA-1273.351: LNP encapsulated mRNA-based vaccine that encodes for a full-length, prefusion stabilized S protein of the SARS-CoV-2 B.1.351 variant	Moderna and National Institute of Allergy and Infectious Diseases (NIAID)	Phase 4	No report yet	-
	LNP-nCoVsaRNA	Imperial College London	Phase 1	No report yet (seroconversion at week six was related to dose, ranging from 8% (3/39; 0.1 μg) to 61% (14/23; 10.0 μg))	[48]
	BNT162b2 (three LNP-mRNAs), also known as ‘Comirnaty’	Pfizer/BioNTech and Fosun Pharma	Phase 4	91.3% (CI 89.0–93.2)—2 doses	[49]
	LNP-nCOV saRNA-02 vaccine: saRNA encapsulated in LNPs	MRC/UVRI and LSHTM Uganda Research Unit	Phase 1	No report yet	-
	mRNA-1273.211: multivalent booster candidate combining mRNA-1273 plus mRNA-1273.351	Moderna TX, Inc.	Phase 2/3	No report yet	-

## NANOTECHNOLOGY FOR THE MANAGEMENT OF COVID-19

**Figure 3.**
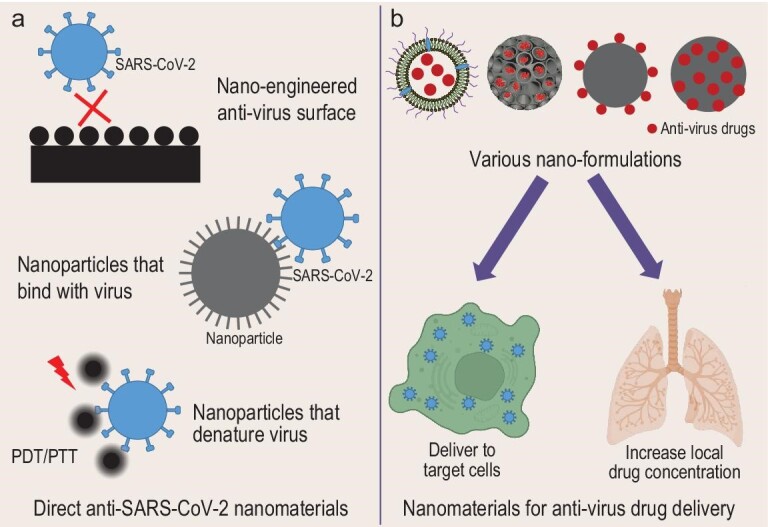
Nanotechnologies for the management of COVID-19. (a) Nanotechnologies that fight directly against SARS-CoV-2. (b) Nanomaterials as drug delivery systems against SARS-CoV-2.

Unlike traditional therapeutics, which tend to target a specific viral species and may lose their efficacy as the virus accumulates mutations, antiviral nanomaterials can target many types of viruses, because of their customized chemical and physical properties. DNA-based nanostructures can trap viruses, while modified polymers can serve as cell membrane decoys. Other nanomaterials can disrupt viral envelopes. Using such approaches may offer advantages in the context of countermeasures in a pandemic, as they can be formulated rapidly (Fig. 3).

### Nanomaterials with direct anti-SARS-CoV-2 activity

Although SARS-CoV-2 can be transmitted readily by droplets and aerosols, contact transmission is an important additional route, via contaminated inanimate surfaces and poor hand hygiene [50]. The virus SARS-CoV-2 remains active on a range of surfaces, including glass, metal, wood and plastic, for up to several days [51]. As a result, there is interest in using antiviral nanomaterials [52,53] and nanoparticles that can carry antiviral agents [54]. Antiviral activity against SARS-CoV-2 has been shown for a number of nanoparticles, including silver [55], zinc oxide [56], cuprous oxide [57], silica, gold [58] and graphene oxide grafted with metal nanoparticles [59]. When used to coat personal protective equipment, the combination of graphene oxide with silver, iron, zinc and copper nanoparticles improves antiviral activity against both enveloped and non-enveloped viruses [59].

Given the importance of respiratory protection via surgical respirators for health care workers during the pandemic, there has been interest in using such composite nanomaterials to coat the fibres within the respirators, or on the outer surface of the filtering facepiece. Study has demonstrated that a silver nanocluster/silica composite coated respirator has an effect against SARS-CoV-2 [60], with the presence of silver on fibres confirmed using energy dispersive spectroscopy. Such coatings could be deposited on polymeric, metallic and glass surfaces.

A further method for providing a smart antimicrobial surface is to use nanomaterials that are light responsive [61], to achieve disinfecting actions after activation by specific light wavelengths through photodynamic, photothermal or photocatalytic processes [62,63]. As an example, a nanoscale coating of a TiO_2_ photocatalyst can inactivate SARS-CoV-2 on the surface when there is exposure to light [61]. Nanotechnology can also improve conventional disinfection methods, and overcome the limitations of common biocides (such as ethanol and sodium hypochlorite) when levels of surface protein bioburden are high [64]. Several approaches have been introduced thus far. Temperature-responsive antimicrobial nano-coatings can provide a prolonged disinfecting action by damaging the envelope of the virus, in response to applied heat.

Nanomaterials can target different stages of SARS-CoV-2 viral infection, such as fusion of the virus with the host cell membrane, internalization, viral genome transcription, translation and replication. Nanomaterials can be designed to trap and neutralize SARS-CoV-2 with high efficiency. For example, Nie *et al*. prepared silica nanoparticles with 5 to 10 nm spikes, which could be inserted into the surface glycoproteins of the influenza A virus and neutralize them [65]. It is likely that nanomaterials with various combinations of geometry-matching topography and virus-binding sites will be developed to target SARS-CoV-2 as well as other viruses.

Photothermal antiviral actions can be achieved rapidly when nanoparticles absorb near-infrared light, and the resulting heat causes photothermal disinfection [66]. The heat generated will denature pathogens by damaging envelopes or nucleic acids, or by denaturing enzymes [67,68]. As an example, Miyako *et al.* have described PEG-carbon nano-horns tagged by an antibody that specifically targets the T7 bacteriophage, and binds the nano-horns to the virus [53]. Irradiation of the attached nanoparticles with near-infrared light from an Nd : YAG laser (wavelength 1064 nm) generates heat and causes photothermal inactivation of the bacteriophage effect. Such functionalized nanoparticles can also be effective against other viruses, including HIV, influenza and SARS-CoV [53]. Another light-to-heat conversion platform with powerful photothermal disinfecting actions features sulfonated magnetic nanoparticles that have been functionalized with reduced graphene oxide. When these nanomaterials were irradiated by near-infrared laser light (808 nm, 1.6 W/cm^2^ for 10 min), the temperature rose to 55°C, and this inactivated 99.99% of type-1 herpes simplex virus [69,70].

A further photothermal process uses gold nanorods that have been functionalized with angiotensin-converting enzyme-2, which will bind SARS-CoV-2. These particles can be activated by near-infrared laser irradiation (798 nm), to achieve selective elimination of SARS-CoV-2. This type of approach may have value when patients are hospitalized with severe pulmonary infections [71]. Several major concerns would need to be addressed before such clinical applications of photothermal disinfection using nanoparticles could be contemplated, including the safety and toxicity of the nanorods, and the extent of bystander thermal effects on host tissues.

Compared to traditional antiviral drugs, which usually work only for specific viruses, nanomaterials may provide broader targeted virus ranges as the antiviral effects are mainly from their chemical and physical properties. For example, the spiky silica nanoparticles may work for the mutations of the viruses as well as bind to the glycoproteins of the virus with their nano-topography [65]. Nanomaterial-based strategies can provide fast and cheap tools for the management of pandemics, as they can be developed quickly and work against a large range of viruses including their variants.

### Nanomaterial-based drug delivery for managing SARS-CoV-2 infection

Nanomaterials can be used as smart carriers in targeted drug delivery systems for antiviral medicines. This leverages their distinct properties, including large surface area, good biocompatibility and ease of surface modification, all of which can be customized during design [72]. Using a nanotechnology approach may overcome challenges with antiviral medicines, such as poor aqueous solubility and low bioavailability. It may also lower the doses needed, and thereby reduce toxicity [73,74]. It is also possible to use nanoparticles to target specific organs or cells that are involved in the pathophysiology of the infection [75]. In addition to small molecular drugs, nanoparticles can also effectively deliver other bioactive molecules, such as nucleic acids, proteins and peptides [76]. Various types of nanoparticles, including inorganic nanoparticles such as metals and organic nanoparticles such as lipid/liposome, and polymer nanoparticles, have been explored for antiviral drug delivery, including anti-SARS-CoV-2 drugs.

Inorganic nanoparticles with small size (1 to 100 nm) such as metal nanoparticles can be synthesized, and the corresponding increase in the surface area gives them a high loading capacity for antiviral agents [77]. Several metal nanoparticles have been investigated for antiviral therapy. For example, selenium nanoparticles have been used to deliver several antiviral medicines, including ribavirin, oseltamivir and zanamivir, to prevent apoptosis induced by H1N1 strains of the human influenza virus [78]. Since ribavirin shows some effects against coronaviruses, including SARS-CoV and MERS-CoV [79,80], using selenium nanoparticles to carry ribavirin may also have value in the treatment of SARS-CoV-2 infections. Gold nanoparticles can also be used to deliver ribavirin. This approach has been used in cell cultures with measles viral infections in an African green monkey cell line. Using the gold nanoparticles as carriers improves the effectiveness >5-fold [77]. Gold nanoparticles with long linkages of mercaptoethanesulfonic acid and sulfonate undecanesulfonic acid can reduce membrane fusion caused by MERS-CoV [81], making this of interest for SARS-CoV-2 treatment. In addition to selenium and gold, other nanoparticles, including silver [82], mesoporous silica [83,84] and iron oxide [85], are prospective candidates for delivering antiviral medicines. In addition to metal nanoparticles, cyclodextrin-functionalized multi-walled carbon nanotubes have been used successfully to treat herpes simplex viral infection through the sustained release of acyclovir [86].

Potential concerns with metal nanoparticles include poor biodegradability, with attendant risks of accumulation within organs. Lipid-based nanoparticles are attractive for clinical use because of their good biocompatibility and biodegradability. Lipid nanoparticles have been used as nanocarriers for antiviral agents, for treating hepatitis C and B, herpes simplex and HIV [87,88]. Liposomes can be used to deliver both hydrophobic and hydrophilic agents. Liposomes containing acyclovir applied via the intranasal route achieve greater bioavailability for the drug (by 60%) compared with intravenous administration [89]. Lipid coating of other nanoparticles, such as mesoporous silica nanoparticles, can also be undertaken to enhance biocompatibility and duration of action in circulation *in vivo*, and to improve efficiency [90].

Polymeric nanoparticles have attracted interest because of the considerable flexibility of their design and ease of modification. Poly (lactic-co-glycolic acid) (PLGA) nanocarriers have been shown to boost the antiviral actions of dyphylline in H1N1 influenza infections, because of the sustained release of the drug in the lung. Optimized PEGylation of these nanoparticles prevented activation of macrophages in the lung for four weeks [91,92]. Such immune modulation effects of nanoparticles could potentially be useful when treating severe SARS-CoV-2 infections.

Polymeric nanoparticles can be loaded with corticosteroids and inhaled. This approach has been used to treat asthma and severe chronic obstructive pulmonary disease, but could also be applied to SARS-CoV-2 infection in patients experiencing severe illness because of a cytokine storm. Multifunctional polymeric nano-delivery systems have considerable potential for clinical application, and with emergency or expedited approval could be deployed in the current COVID-19 pandemic [93].

Dendrimers are synthetic nanostructures with a well-defined branching architecture. They have good biocompatibility, high solubility and are effective for drug encapsulation. Previous works have shown their efficiency as a delivery system in the context of herpes simplex virus type-2, HIV and influenza [94], and dendrimer nanoparticles are tested for the management of SARS-CoV-2 [5].

## NANOTECHNOLOGY FOR THE POST-PANDEMIC ERA

With several highly effective COVID-19 vaccines deployed at scale in many countries, an important question is whether the use of nanotechnology in vaccines provides the necessary ability to rapidly redesign vaccines when concerning variants appear that are more virulent. Outlined below are the major advantages of mRNA delivered in lipid nano-carriers.

### Nanoscale information in the battle with SARS-CoV-2 variants

As SARS-CoV-2 continues to mutate, new variants of concern are being generated with altered virulence and transmissibility, resulting in altered levels of protection from existing vaccines [95]. The Omicron variant has shown a particularly rapid global spread and has replaced Delta as the most prevalent variant. With the help of nanotechnology, scientists determined the nano structure of the Omicron spike protein in complex with human ACE2 at 2.45 Å resolution using Cryo-electron microscopy [96]. This study also revealed the strong interaction between the mutated spike protein and ACEs due to the forming of new salt bridges and hydrogen bonds, which explains the rapid spread and increase in antibody evasion of the Omicron variant [96]. This nanoscale information, and the new understanding based on that nanoscale information, will help us to limit the spread of Omicron and other potential variants.

### Nanotechnology that enhances the global distribution of vaccines

Lipid nanoparticles that contain mRNA have been proven to be an efficient method for developing immunity. Low or ultra-low temperature storage of these lipid nanoparticle-mRNA vaccine formulations (−20 or −80°C) is not convenient for shipping, storage and distribution, especially in developing countries and in remote areas. A new thermostable lipid-nanoparticle-encapsulated mRNA (mRNA-LNP) vaccine, known as ARCoV, has been developed by a modified fabrication process. ARCoV was manufactured through rapid mixing of mRNA in an aqueous solution and a mixture of lipids in ethanol, followed by tangential flow filtration to remove ethanol and concentrate the solution. ARCoV particles are solid spheres without an aqueous core and can be stored at room temperature for at least one week without losing activity. The mRNA encodes the receptor-binding domain (RBD) of SARS-CoV-2. ARCoV is currently being evaluated in phase one clinical trials [97].

Another nanotechnology approach of interest is the so-called ‘nano patch’. This comprises arrays of densely packed projections with a defined geometry, which can penetrate through the epidermis painlessly. Using a patch rather than an injection delivers the vaccines to thousands of antigen-presenting cells in the dermis and epidermis. A nano patch vaccine can be kept at room temperature without needing to be refrigerated. This approach overcomes storage issues and also avoids the need for injections, tackling two barriers at the same time.

### Nanotechnology in preparations for the next pandemic

#### Development of vaccines and nanomaterials for vaccine delivery

The widespread use of lipid nanoparticles to deliver mRNA vaccines has increased the awareness of this platform. The relative ease of production makes this appealing for ‘first response’ vaccines for future influenza or coronavirus pandemics. Lipid nanoparticles could also be used to deliver DNA gene therapies and CRISPR gene-editing therapies.

In addition, other polymer nanoparticles, protein-based nanoparticles, inorganic nanoparticles and exosomes are also worth considering as vehicles for future vaccines.

#### Development of antiviral nanomaterials

As already discussed, nanoparticles can deliver antiviral medicines and can also, in some cases, exert their own antiviral actions against multiple viruses. This makes them rather different from traditional antiviral agents, which have a limited range of targets. Many viruses, including SARS-CoV-2, rely on glycoproteins on their surface to bind to and then enter host cells. Nanomaterials can be designed to mimic binding sites. As an example, Zhang *et al*. prepared ‘nanosponges’ that displayed the same protein receptors as human cells and showed that these can bind to and neutralize the SARS-CoV-2 virus, preventing it from infecting cells. This nanosponge approach is not expected to be affected by viral mutations [98].

## CONCLUSIONS AND OUTLOOK

Nanotechnology has empowered the global response to the COVID-19 pandemic, through powerful tools for prevention, diagnosis and treatment. Detection systems based on nanoparticles and nanopores have enabled rapid and inexpensive detection of the virus, and have informed public health measures. One lesson we have learned during this COVID-19 pandemic is that rapid, large-scale virus detection can greatly help disease control and requires the development of virus detection methods that are simple to use, and have high accuracy and low cost. The power of nanotechnology-driven detection such as the LFA and nanopore sequence can be further explored to manage COVID-19 and other potential virus diseases.

During this pandemic, lipid nanoparticles for delivering mRNA in vaccines have played a major role in population-level vaccination strategies, and will likely play an increasing role in the future, both as a platform for the rapid development of vaccines, and for updating vaccines to address viral mutations. New nanoparticles with higher antigen-delivery efficiency, better stability, especially thermal stability, and target delivery are desired for vaccines. This ability to adjust to the challenges posed by a rapidly mutating virus is a major advantage of nanotechnology. Nanomaterials that have potent antiviral actions also have considerable promise.

In the long term, nanotechnology will serve as a technological foundation for the prevention and management of future viral-infection pandemics. Appreciation of the opportunities that nanotechnologies offer is necessary for effective collaboration between scientists, policymakers and health care professionals when addressing the long-term challenges caused by SARS-CoV-2 and potential virus outbreaks.
